# Converging Evidence of Ubiquitous Male Bias in Human Sex Perception

**DOI:** 10.1371/journal.pone.0148623

**Published:** 2016-02-09

**Authors:** Justin Gaetano, Rick van der Zwan, Matthew Oxner, William G. Hayward, Natalie Doring, Duncan Blair, Anna Brooks

**Affiliations:** 1 Cognitive Neuroscience Research Cluster, Southern Cross University, Coffs Harbour, Australia; 2 Department of Psychology, University of Hong Kong, Hong Kong, People’s Republic of China; Duke University, UNITED STATES

## Abstract

Visually judging the sex of another can be achieved easily in most social encounters. When the signals that inform such judgements are weak (e.g. outdoors at night), observers tend to expect the presence of males–an expectation that may facilitate survival-critical decisions under uncertainty. The present aim was to examine whether this *male bias* depends on expertise. To that end, Caucasian and Asian observers targeted female and male hand images that were either the *same or different* to the observers’ race (i.e. long term experience was varied) while concurrently, the proportion of targets changed across presentation blocks (i.e. short term experience change). It was thus found that: (i) observers of own-race stimuli were more likely to report the presence of males *and* absence of females, however (ii) observers of other-race stimuli–while still tending to accept stimuli as male–were not prone to rejecting female cues. Finally, (iii) male-biased measures did not track the relative frequency of targets or lures, disputing the notion that male bias derives from prior expectation about the number of male exemplars in a set. Findings are discussed in concert with the *pan-stimulus* model of human sex perception.

## Introduction

The ability to process sex cues pre-dates the emergence of human civilisation, and to present date is an important mediator of social interaction. Exposure to sexually dimorphic cues can subtly prime an individual’s mood [1], moderate physiological responses [2, 3], and even sway life-critical decisions [4]. Despite sex perception’s importance to both our species’ survival and to everyday human behaviour, scientific explanations of its bases are still in their infancy and tend to focus on but one set of dimorphic features: the face.

What little is known about the cortical correlates of sex perception suggests an involvement of brain regions *not* specially selective for faces. This dissociation between face processing per se, and face-based sex processing has been observed in two ways. Firstly, in healthy adult brains, sex selective neural activity seems to occur in regions that do not overlap perfectly with face specialised regions [5–7]. Secondly, face-based sex categorisation ability is preserved in otherwise ‘face-blind’ patients [8–10]. Taken together, the data suggest at the very least that neural correlates of sex perception are not a simple subset of those associated with face recognition.

In line with those observations, some behavioural correlates of sex perception seem to involve mechanisms that range beyond face-based cues to include *hands*. Hands have been shown to be a useful sex signal, and subject to certain effects similar to those observed for faces [11, 12]. One of those is the *upper-visual field advantage*: Sex categorisations are more easily disrupted by distractors when targets are presented below fixation (faces: [13]; hands: [14]). Similarly, age aftereffects transmit between hands and faces [15]. In other words, processes mediating sex perceptions from face-based cues manifest also when using hands as sex cues. Moreover, the processes handling faces and hands interact, giving rise to an interesting question: are the effects described above the outputs of a single sex cue processor that operates on both types of sexually dimorphic cues, or are there several processors?

To help answer that question, the current study explored another class of phenomena associated with face, and now hand processing: observer bias. Eye-tracking studies have shown that Caucasians and Asians tend to focus on different facial regions [16–18] and that those fixation biases are maintained for inverted faces [19]. Similarly, Asian and Caucasian observers have developed different attentional strategies for own-race versus other-race faces [20, 21]. Although those same attentional differences have not been shown for hands, one effect that is common to both face and hand sex discriminations is *male bias* (MB). In perceptually ‘noisy’ environments–those in which difficulty in discriminating female from male is high–observers tend to report ambiguous cues as being male. Specifically, such biases can occur when there is natural [22, 23] or artificial [24, 25] sex cue weakness in terms of signal to noise ratio, or a weakness imposed by lack of inspection time [12, 26].

Whilst MB seemingly reflects a failing of perception under uncertainty, it could in fact serve an adaptive purpose; one of biological risk detection and management. Males are on average physically stronger and more prone to violent behaviour [27, 28] than are females, and thus in ambiguous environs (e.g. a poorly lit street at night) they may represent a larger potential threat to personal safety. The ability to forestall and prepare for a fight or flight scenario is, therefore, enhanced by guessing that an unknown other is male. Alternatively, guessing ‘female’ is relatively costly: the decision to approach a mysterious figure because of this hunch is–if it is incorrect–potentially dangerous. Such asymmetry in risk has been empirically linked to male-biased response strategies before [29–31], including the decision to shoot to kill [4]. Under ambiguous conditions, even slight cues to maleness can override the reception of objectively stronger, female signals. For instance, observers in Johnson, Iida, and Tassinary’s study [29] categorised all but the most exclusively female body morphs in a male-biased manner. The authors argued that the perceived association between maleness and formidability, coupled with the human instinct to protect oneself from harm, explains the purpose of MB. Expanding on this, stimuli that are not just formidable but also *foreign* to the observer could amplify the bias to prepare for threatening males. Other-race faces do indeed resist fear extinction relative to own-race faces [32–34], and compellingly, Navarrete et al. [3] found that this fear extinction bias occurs *only* for male stimuli. If MB does perform an *adaptive* function (e.g. risk avoidance under uncertainty), then the effect should generalise to ambiguous situations wherein females and males can be viewed. In fact, MB seems to *not* depend on any particular stimulus type. It can manifest when judging sex from faces [24, 26], hands [12], and whole bodies (structure: [22, 29]; movement: [23, 25, 35]). If there is a single sex processor that functions independent of cue type, then cross-race effects should be observed too in MB for hands–just as there are cross-race effects observed for faces.

Gaetano et al. [12] have showed that MB is not dependent on hand targets being male (i.e. observers asked to identify *females* do so conservatively). More specifically, it was suggested that MB arises as the consequence of a dual-criterion shift. When instructed to search for a target, observers *both* (i) tighten their criteria for inclusion into the target category when searching for females, and (ii) relax their criteria for inclusion into the target category when searching for males [12]. This suggests that MB is not a (cognitive) response bias associated with preference for the label ‘male’ (cf. [26]). In addition, and like many perceptual phenomena, the MB seems also dependent on experience. Wild and her colleagues found that MB was higher for younger observers [26], and on that basis she suggested that processing experience might partially decrease the bias (cf. [36]). If that is true, own-race expertise should mediate the MB.

Stimulus independent interactions between MB and stimulus familiarity would imply the existence of sex processing mechanisms that are tuned by signal strength *and* experience. Wild et al.’s [26] findings have demonstrated such interaction–when cues to sex were weak, observers adopted male-biased response criteria, especially if they were perceptually inexperienced (i.e. children versus adults). Aside from prior experience *differences* on the scale of decades, varying experience on more proximate scales may also change the expression of MB. Indeed, sex judgements of targets can be biased by contextual or environmental cues [36, 37], sometimes without awareness on the observer’s part [38]. What has not yet been tested is whether a target’s perceived sex might be influenced by changes to *target* prevalence across trials. With that in mind, prior target probability (PTP) was considered in the present study as a means to investigate the flexibility of MB for own- versus other-race hand stimuli. If MB is labile in the short term, it should change online in response to the relative availability of female or male cues. If however the effect is a heuristic associated with rigid *expectations* about the proportion of female and male cues, then male-biased criteria should appear to wax and wane linearly as the actual proportion changes.

Clues regarding the likelihood of PTP-mediated MB can be found in recent works on biological perception. One such work is a study by Troje et al. [25], in which observers demonstrated a tendency to perceive *objectively androgynous* point-light walkers as male. In contrast, the *subjectively* sex-neutral walker contained more female than male cues. Hoping to cancel out this MB, the authors’ subsequently centred the range of morph stimuli on the subjectively neutral walker, which nevertheless prompted observers to MB their criteria *even further* [25]. More recently, Dahl, Chen, and Rasch [39] found that changing the frequency of more familiar (own-race or -species) relative to less familiar (other-race or -species) stimuli impacted upon discrimination performance for either category. Effectively, the authors [39] have demonstrated that own-race and own-species advantages–two effects commonly explained in terms of perceptual expertise developed over a lifetime (e.g. [40, 41])–can be counteracted by increased exposure to *less familiar stimuli in the short term*. In the present study then, MB across races might be subject to short term stimulus set effects–it might show to be labile in the face of immediate experience.

Accordingly, the general aim of this work was to test the MB for long- and short term experience invariance. It was predicted that given ambiguous viewing conditions, observers would: (i) categorise sex from hands in a male-biased manner, irrespective of observer or hand race; (ii) adopt both a strict criterion when assigning a target as female, and a loose criterion when assigning a target as male (i.e. the *dual-criterion hypothesis*), and; (iii) set those criteria independent of live changes to target probability. In summary, the dual-criterion hypothesis was found to depend on observer expertise: own-race observers’ criteria for ambiguous male and female targets were respectively ‘yes’ and ‘no’ biased, whilst other-race observers were biased to accept males but not to reject females. Furthermore, group MB measures did not predictably vary as a function of PTP.

## Methods

### Ethics statement

All observers gave written, informed consent prior to participating in the study. All experiments were approved by the Human Research Ethics Committee, SCU (Approval numbers: ECN-11-236; ECN-12-280; ECN-13-032; ECN-14-028). In addition, all experiments conducted in Hong Kong were approved by the Human Research Ethics Committee for Non-Clinical Faculties, University of Hong Kong. This study complies with the ethical standards specified by the Declaration of Helsinki.

### Observers and stimuli

Eighty Caucasians (47 female) and 80 Asians (39 female) took part in this study. Caucasians were on average older (*M* = 32.49, *SD* = 11.08) than were Asians (*M* = 21.50, *SD* = 2.72), and this difference was found to be significant (*F*_1,157_ = 72.50, *p* < .001). Thus, the potentially mediating effects of observer age on response criteria was investigated in combination with two other unplanned factors: observer race and sex. If response criteria was not overall influenced by the selected observer variables, then ANCOVA will reveal no significant main and/or interaction effects. The same analyses were run independently for each presentation duration (1000 ms and 125 ms) and racial familiarity group (own-race and other-race); and at each level of ambiguity (low [colour], high [silhouette]; α = .05 per level or group). Race and sex formed the between-group factors, and age was included as a covariate. All output data are available in S1 Dataset. To summarise, observer ages did not covary with response criteria. With age thus controlled for, overall it was found that observer race, observer sex, and observer race × sex did not affect response criteria. Therefore, in subsequent analyses, observer age was ignored and data were collapsed across race and sex groups.

**Fig 1:**
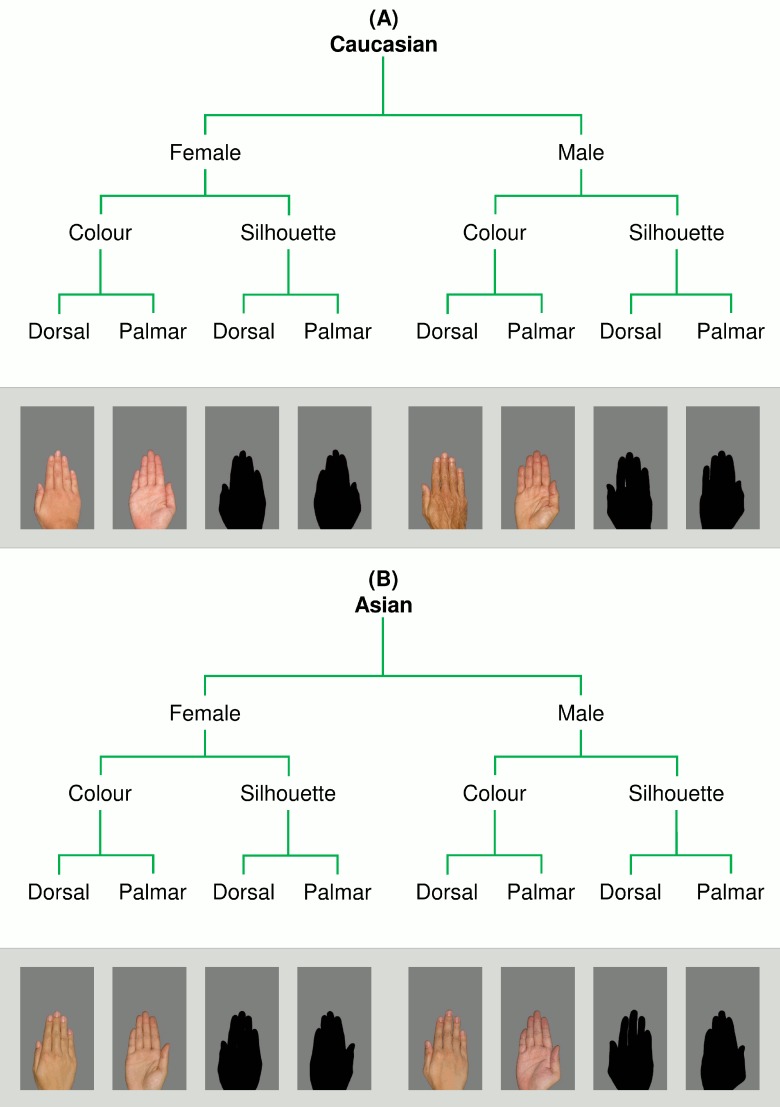
Stimulus exemplars used in the current study. (**A**) Caucasian and (**B**) Asian hand stimuli were reduced to the size of the smallest Caucasian exemplar without altering their natural aspect ratios. Within each stimulus condition 15 female and 15 male exemplars were represented.

Thirty Caucasian (15 female) and 30 Asian (15 female), individual hands formed the basis of the stimulus set used in the present experiment. Stimuli were prepared as per methods developed by Gaetano et al. [12]. Photographs were taken of each individual’s right palmar and dorsal hand surface. Exemplars were reduced to the size (as indexed by total pixel count) of the smallest (female) Caucasian hand (105,069 px at 70.87 px/cm resolution) such that their natural aspect ratios were preserved. Images were presented with all hue and texture information preserved (‘colour’ condition), and also with those cues subtracted (‘silhouette’ condition). Stimulus exemplars are depicted in Fig 1.

### Design

#### Prior target probability

As mentioned above, perceptual expertise for particular (e.g. own-race) cues develops over the long term, which might impinge on how strongly MB is expressed. MB could also be mediated by shorter-term perceptual weighting of particular cues. To test this, bias was measured in synchrony with probabilistic changes to the stimulus sample.

Of course, in a large enough human population, there are approximately equal numbers of females and males [42] such that the optimal (2AFC) sex classification guess rate would be 50%. In the laboratory, it is possible to change PTP (i.e. the sex ratio) and thus redefine optimality across experimental blocks. For instance, the optimal ‘female’ (or ‘male’) guess rate will increase as the ratio of female to male (or vice versa) stimuli increases–when PTP is female-biased, a blindfolded observer’s estimation that any given cue is *probably* female must be closer to optimal than the assumption that half of all cues are female.

**Fig 2:**
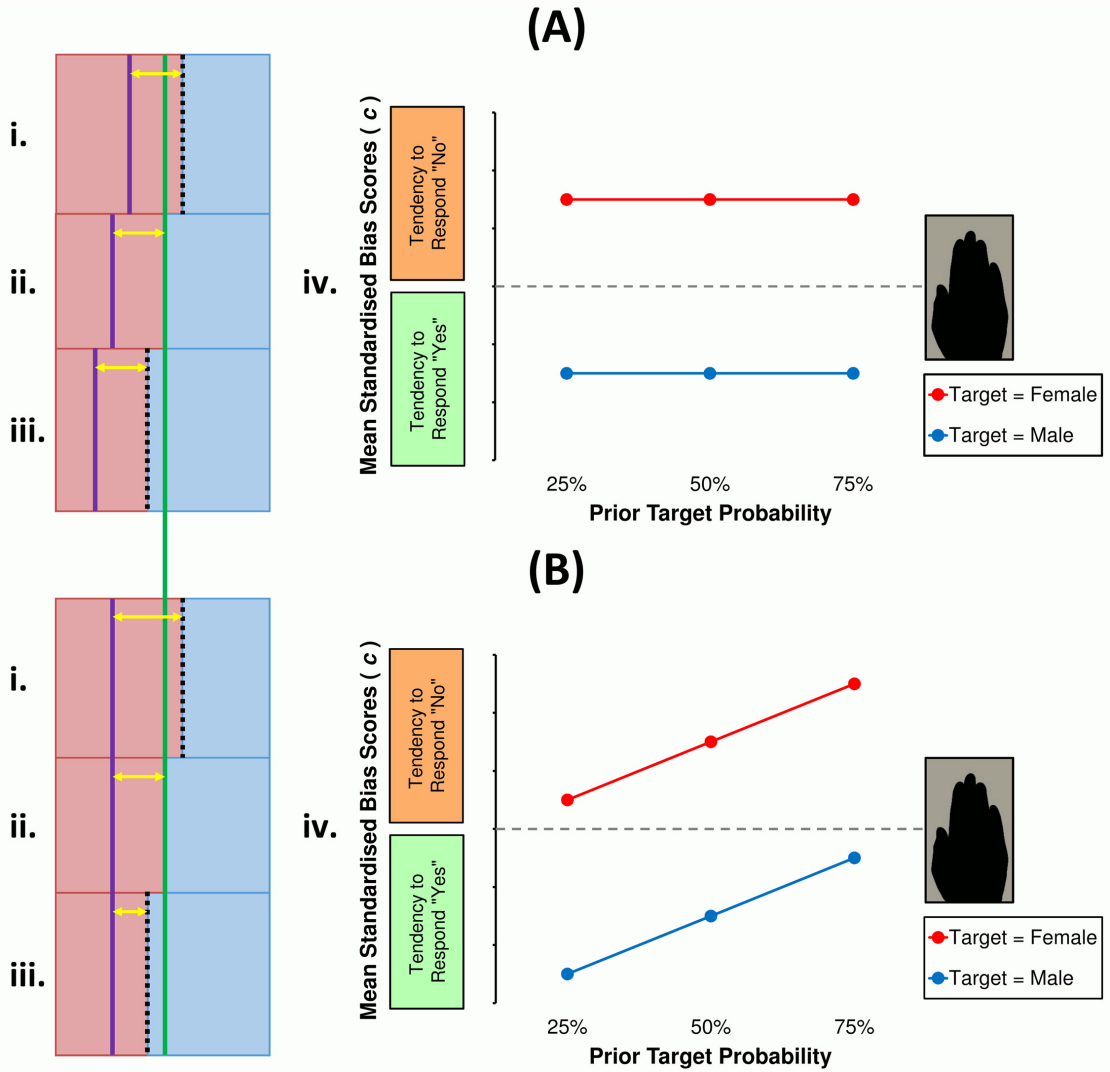
Alternative models and predicted outcomes of male-biased criteria setting. As PTP changes, MB may arise via opposing mechanisms. The size (not to scale) of each blue relative to pink rectangle represents PTP (**i**: 25%; **ii**: 50%; **iii**: 75%). (**A**) If, under ambiguous conditions, observers are nonetheless sensitive to PTP change, they should attenuate their liberal male criterion (**i-iii**: purple lines) relative to the optimal criterion (**i, iii**: dotted lines; **ii**: green line) when targets are (**i**) scarce, (**ii**) equal, or (**iii**) common. If so, in the present study (**iv**) there should be no difference between mean criteria as measured under different PTP conditions. (**B**) Alternatively, if observers neglect change in optimality, they may set male-favouring criteria (**i-iii**: purple line) relative to a fixed population estimate of PTP (green line). This would effectively inflate criteria measures when male targets are scarce (**i**: yellow arrow) and diminish the same when targets are frequent (**iii**: yellow arrow), which in the present study (**iv**) would manifest as a linear association between bias measures and PTP.

Applying this logic to the current design, if MB is a labile or adaptive process, observers will update their guess rate accordingly–they will appear to be male-biased at the same level, irrespective of how many targets or lures there are. In other words, under ambiguous conditions, the ‘male’ identification rates of observers would diminish as female stimuli became prevalent, and increase as male stimuli became scarce, and this would be measured as similar bias levels across those conditions; that ‘labile MB’ hypothesis is depicted in Fig 2A. If, however, MB is a *static* routine, observers will not readjust their already biased criteria in line with changing optimality. That is, under ambiguous circumstances, ‘male’ identification rates would remain fixed given female-biased, non-biased and male-biased stimulus ratios. Thus, a rigid or non-adaptive MB strategy would result in deceptively larger measures of bias when males are *rare*, because they would be responding ‘male’ on predominately female trials. This alternative outcome is represented in Fig 2B.

#### Procedure and analyses

An equal number of Caucasian and Asian observers were assigned randomly to one of two experiments that differed only by stimulus presentation duration (Experiment 1: 1000 ms; Experiment 2: 125 ms). Within each, observers were further divided equally into an *own-race* (i.e. Caucasian/Asian observers of Caucasian/Asian hands) and *other-race* (i.e. Caucasian/Asian observers of Asian/Caucasian hands) group via random allocation. Thus, each experiment recruited 40 Caucasian and 40 Asian, own- or other-race observers.

Each trial comprised in chronological order: a blank screen for 1000 ms, a stimulus presentation (125 ms or 1000 ms), and a response screen (centred cross, +, on black background) that extinguished once a response was made or 1000 ms had passed. At the response screen of each trial, the observer’s task was to indicate via key press whether the image represented a target sex (‘yes’) or not (‘no’). Each image was presented across six blocks–two target sexes (female/not female, male/not male) and three PTPs (25%, 50%, 75%)–for a total of 720 trials per observer. Stimulus order was randomised for each observer. Block order as well as response key alternatives were counterbalanced across observers.

For each observer, standardised criteria scores *c* [43] were calculated as an average on all (palmar and dorsal) trials on each condition of interest. Two sets of analyses were applied independently within each experiment. In the leading between-group analyses, only data from blocks in which PTP was equal (50%) were analysed. Criteria averages were calculated as the absolute mean difference, *c*_*A*_, between target sex condition means (*c*_*A*_ = *c*_*female*_—*c*_*male*_); negative and positive *c*_*A*_ scores represent respectively female- and male-biased response criteria relative to the hypothetical optimal or bias-free observer (*c*_*A*_ = 0.00), expressed in standard deviation units. Orthogonal contrasts [44] were conducted in PSY [45] to determine whether the overall MB rates of Caucasians or Asians differed by expertise (own-race vs. other-race observers), and those were followed up by single-sample *F*-tests.

Subsequent within-group analyses were applied to only the most perceptually impoverished conditions–those *silhouette* conditions wherein hue/texture cues had been eliminated from stimuli. Within each group, mean *c* scores were measured at each level of target sex (female, male) and PTP (25%, 50%, 75%). Unlike *c*_*A*_, negative and positive *c* scores represent respectively the tendency for an observer to respond ‘yes’ or ‘no’ to a target, in this case defined as either ‘female’ or ‘male’. To test whether bias shifted predictably as a function of PTP, linear and quadratic trend analyses were performed [44, 45]. Specifically, performance was contrasted (i) across 25% and 75% PTP conditions and (ii) across those conditions combined and 50% PTP conditions, separately for each level of target sex (female, male). Then, within each group, single-sample *F*-tests (each α = .008) were run on each condition mean. Finally, an effect size estimate accompanied each analysis, irrespective of significance. Eta squared values (η^2^%) were calculated to denote the percent variance explained by each *planned effect*, while Cohen’s *d*_*z*_ represented the standard distance of single-sample means from the hypothesised population mean [46].

## Results

### Individual differences in male bias

Here, the question of exactly *how many* of the 160 observers were, in a general sense, male-biased is considered. This is an important precursor to justify ‘information loss’ associated with averaging in the main stream of analysis–if observers vary greatly with respects to their average criteria for fe/male targets, then further averaging to create *group* scores would underrepresent such individual differences.

**Fig 3:**
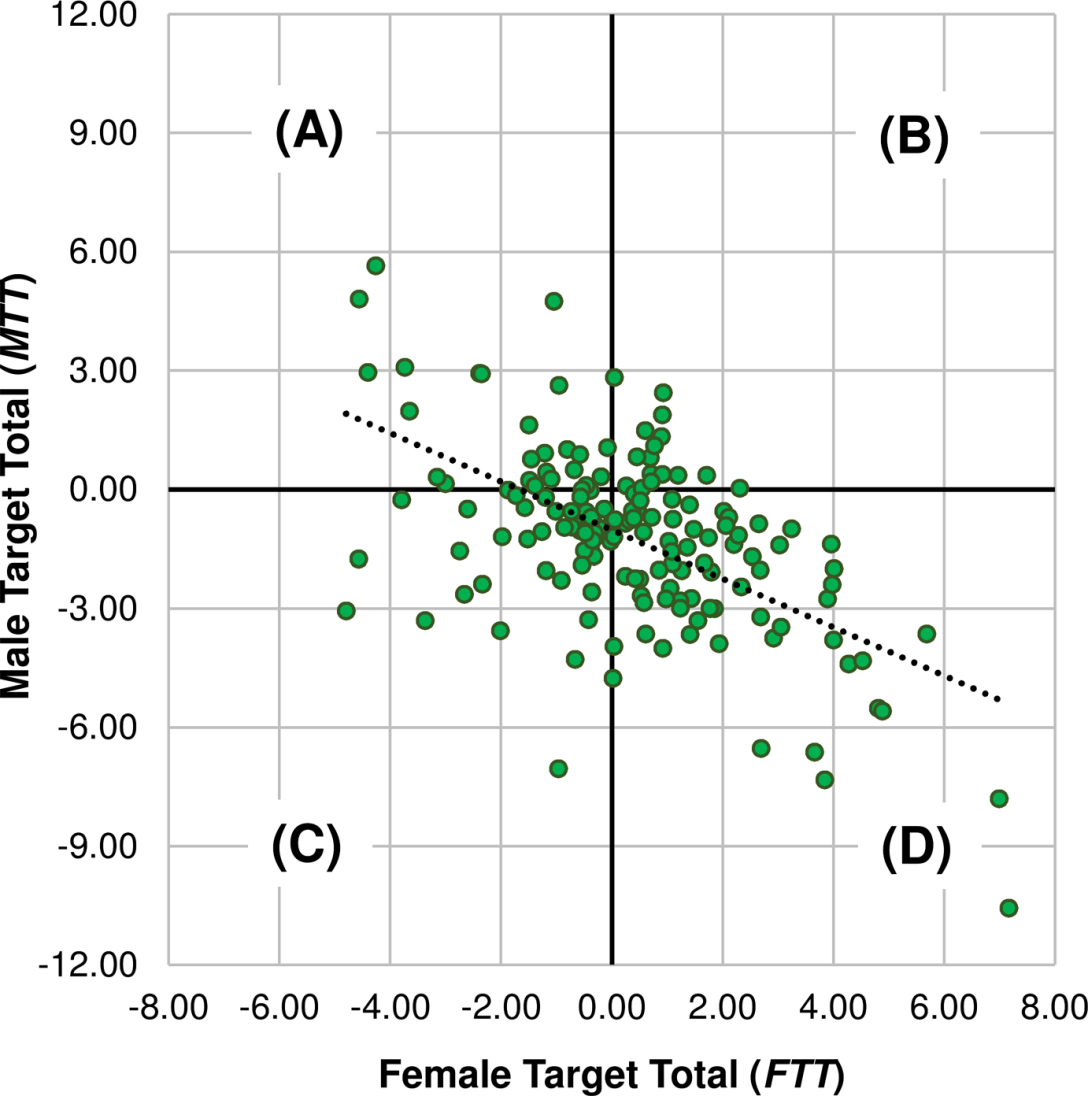
Relationship between two bias totals of 160 hand observers. Observer demographics and statistics are described in the main text. Negative values on either axis represent a tendency to report a target sex as present; positive values indicate a tendency toward reporting target sex as absent. Total bias score from female target conditions (*FTT*) correlated negatively with the score summed from male target conditions (*MTT*) as indicated by the dotted line of best fit. Ignoring differences between conditions or groups (**A**) 16% of observers shared a tendency to report the presence of females and absence of males overall; (**B**) 28% tended to report both the absence of females and males; (**C**) 10% had a tendency to report the presence of females and males and; (**D**) 46% tended toward reporting males as present but females as absent. One data point (x-y coordinates: 7.16, -10.55) is truncated here to preserve scale.

Each observer’s target-specific condition means were summed to calculate two target-specific *bias totals* per observer; a female-target total, *FTT*, and a male-target, *MTT*; both expressed in standard normal units. As with *c*, positive totals indicate a negative (‘no’) response bias and negative totals indicate positive (‘yes’) bias. Fig 3 plots *FTT* against *MTT* for each of the 160 individuals that participated in Experiment 1 and 2. It can be seen from the bottom-right quadrant (Fig 3D) that 74 (46%) observers had both a positive *FTT* and negative *MTT*, indicating a MB totalled across *all* experimental conditions. In contrast, an opposite female-bias grand total was shared by only 26 (16%) observers, who thus had negative *FTT* and positive *MTT* (Fig 3A). The other two quadrants represent the 44 (28%) observers who overall responded ‘yes’ more often (Fig 3C), and the remaining 16 observers (10%) who frequently responded ‘no’ (Fig 3B).

Two McNemar chi-square tests (critical χ^2^ = 5.02 | α = .025) revealed, firstly, that the apparent saturation of male-biased observers versus female- or non-biased observers who participated in this study was significant; χ^2^ = 23.04, *p* < .001; with Φ = 0.38 suggesting a medium-large effect. Secondly, those who were inclined to respond ‘yes’ irrespective of target sex outnumbered significantly those who tended to report the absence of (female or male) targets, χ^2^ = 13.07, *p* < .001, though this effect was smaller than the first, Φ = 0.29. Taking a broader view, Pearson correlation analysis found a significant negative relationship between *FTT* and *MTT*, *r* = -.57, *p* < .001, explaining 33% of the variance in observer bias totals (*r*^2^ = .33). For a given individual then, a conservative ‘no’ rate on female-target trials adequately predicts a corresponding liberal ‘yes’ rate on male-target trials. Finally and perhaps most crucially, each observer’s *MTT* was subtracted from their *FTT* to arrive at a weighted total criterion (*c*_*t*_ = *FTT—MTT*). Thus, positive and negative values of *c*_*t*_ reflect observers who were overall either male- or female-biased, respectively, expressed as standard units of distance from the hypothetical zero point. Consequently, 108 of the 160 observers (68%) deviated from this point in a *male-biased direction*. That is, performance averaged then summed over all experimental conditions was male-biased for most observers. Thus, given the above, readers can be confident that MB is not just a regressive result of *grouping scores across observers* as per the main study’s method.

### Experiment 1

In coherence with the prediction that MB depends on sex cues being difficult to process, it is expected that MB–if it occurs given 1000 ms inspection time–will occur in only the most ambiguous, ‘silhouette’ conditions. A summary of observer data gathered from Experiment 1 is available as S2 Dataset. Bias in general is predicted to be less here than in Experiment 2, wherein perceptual difficulty is increased by limiting presentation duration to 125 ms.

#### Between-group analyses

**Fig 4:**
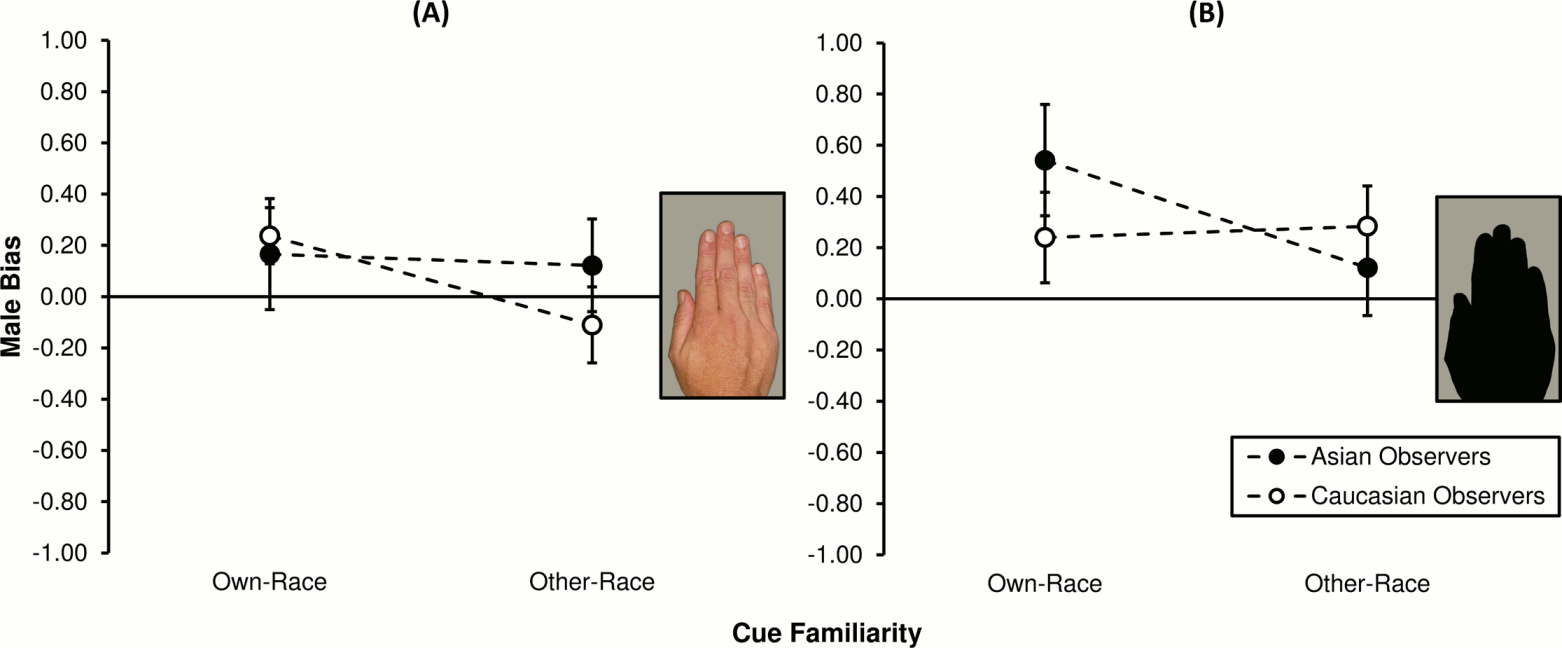
Sex classification bias for observers of hands each presented for 1000 ms. Values on the ordinate represent, in standard units, difference between female target and male target criteria means (*c*_*A*_: see Methods). Scores are grouped by observer race (Asians or Caucasians) and cue familiarity (own-race or other-race hands), plotted separately for the (**A**) intact and (**B**) degraded visual cue condition. Vertical bars represent ±1 *SEM*.

Absolute MB statistics (*c*_*A*_: *M* ± *SE*) were calculated as a function of observer race and familiarity, as plotted in Fig 4. In panel A, one can see that observers of own-race colour hands (Asians: 0.17 ± 0.22; Caucasians: 0.24 ± 0.11) were more male-biased on average compared to other-race colour observers (Asians: 0.12 ± 0.18; Caucasians: -0.11 ± 0.15). This pattern of results persisted somewhat when cues were less obvious (Fig 4B), but seemed to depend on observer race: Own-race Asian MB rates (0.54 ± 0.22) were smaller than for other-race Asians (0.12 ± 0.19), yet the reverse trend was observed for Caucasians (own-race: 0.24 ± 0.18; other-race: 0.28 ± 0.16).

Planned, between-group contrasts showed that cue familiarity did not mediate MB. Specifically, in the colour condition, neither Asian (*F*_1,152_ = 0.03, *p* = .872, η^2^ < 1%) nor Caucasian group MB (*F*_1,152_ = 1.61, *p* = .207, η^2^ = 1%) was found to have differed by experience (own-race vs. other-race cue observers). Likewise in the silhouette condition, MB was no different across own- and other-race observers, independent of their race (Asian: *F*_1,152_ = 2.28, *p* = .133, η^2^ = 1%; Caucasian: *F*_1,152_ = 0.03, *p* = .875, η^2^ < 1%). Thus, the bias means were collapsed by observer race and compared to the level expected under the null hypothesis (*c*_*A*_ = 0.00). Single-sample *F*-tests revealed that neither the own- (*F*_1,39_ = 2.84, *p* = .100, *d*_*z*_ = 0.27) nor other-race (*F*_1,39_ < 0.01, *p* = .959, *d*_*z*_ = 0.01) colour hand mean separated from zero. As for overall performance under the silhouette condition, own-race observers were significantly male-biased (*F*_1,39_ = 7.73, *p* = .008, *d*_*z*_ = 0.44), yet significance was not reached for the other-race group (*F*_1,39_ = 2.79, *p* = .103, *d*_*z*_ = 0.26).

#### Within-group analyses

**Fig 5:**
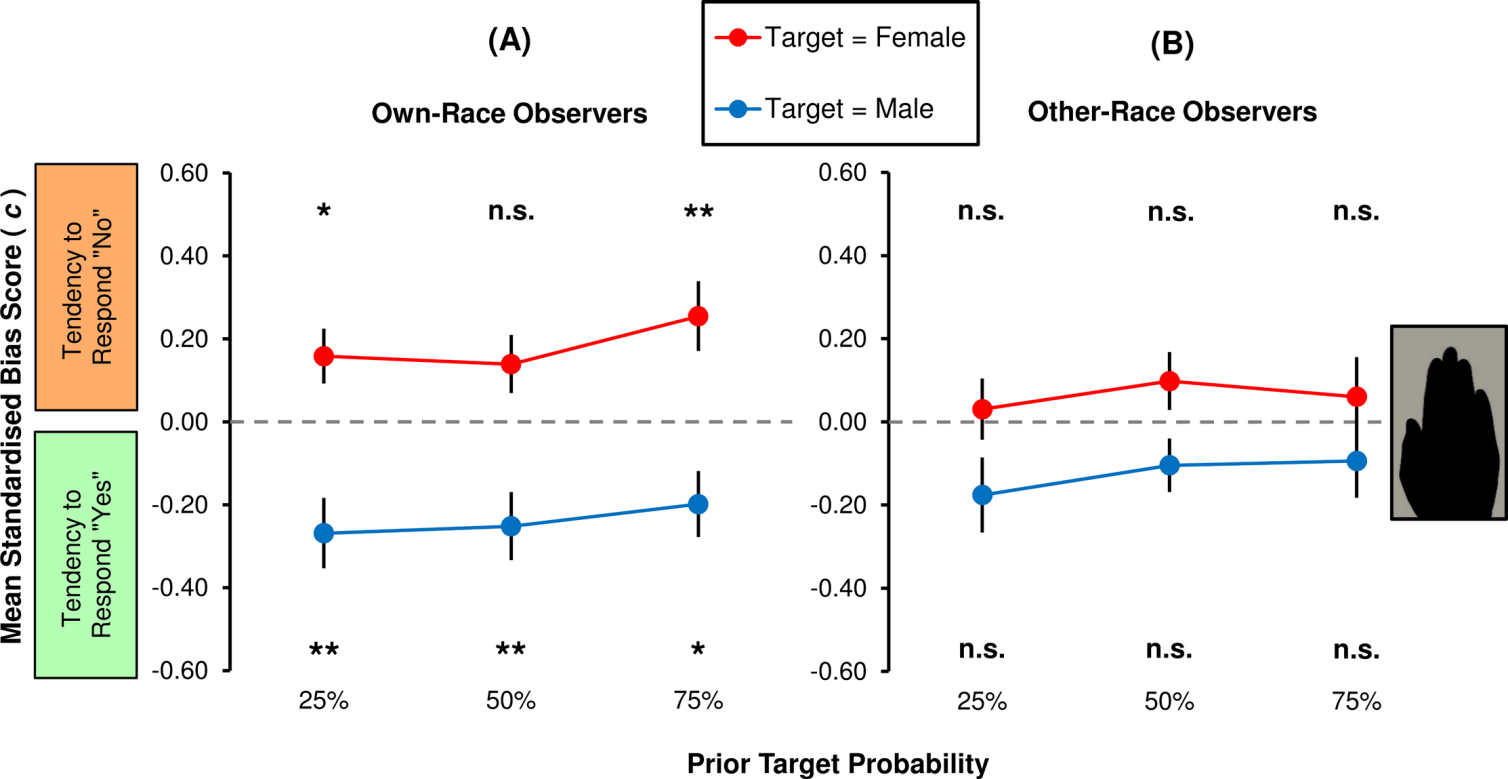
Group bias rates for extended (1000 ms) presentations of silhouette hands. Performances in the ambiguous ‘silhouette’ condition are plotted separately for (**A**) own-race observers and (**B**) other-race observers. Means denoted ** differ from *c* = 0.00 at the .01 level of significance. Means denoted * and ‘n.s.’ correspond to significance values .01 < *p* < .05 and *p* > .05, respectively, and therefore are not significant at the per-comparison criterion of α = .008. Vertical bars represent ±1 *SEM*.

To further break down MB, the absolute scores from the silhouette conditions (as seen in Fig 4B) were separated out into target sex-specific measures of *c*. Within each group of interest, effects of PTP and target sex were analysed respectively via planned comparisons and single-sample *F*-tests. A visual summary of the group statistics are given in Fig 5.

Fig 5A shows the bias statistics relating to *own-race* observers of 1000 ms stimuli. When asked to target female hands, observers did so conservatively. Specifically, they were less inclined to report presence of females when those were *common* (PTP = 75%: 0.25 ± 0.08) relative to equiprobable (PTP = 50%: 0.14 ± 0.07) or rare (PTP = 25%: 0.16 ± 0.07). When the task was to identify males, observers were comparatively liberal. This was especially evident under conditions of fewer (25%: -0.27 ± 0.08) or equal (50%: -0.25 ± 0.08) target trials, and less so when targets were relatively common (75%: -0.20 ± 0.08).

Subsequent tests revealed that such fluctuations in own-race bias across PTP conditions were negligible. When observers searched for female hands, their conservative criterion did not change as a linear (*F*_1,39_ = 1.06, *p* = .311, η^2^ = 3%) or quadratic (*F*_1,39_ = 0.11, *p* = .745, η^2^ < 1%) function of PTP. Similarly in the male target condition, no significant linear (*F*_1,39_ = 2.11, *p* = .154, η^2^ = 5%) or quadratic (*F*_1,39_ = 2.28, *p* = .139, η^2^ = 6%) trend was recovered from PTP condition means. Finally, it was shown that the dual-criterion MB depended on the proportion of targets per stimulus block. When targets were scarce (PTP = 25%), observers were conservative discriminators of females, though not statistically so at the α = .008 level (*c*, female targets: *F*_1,39_ = 5.73, *p* = .022, *d*_*z*_ = 0.38). Also at this level of PTP, observers were significantly liberal discriminators of males (*c*, male targets: *F*_1,39_ = 10.07, *p* = .003, *d*_*z*_ = 0.50). By contrast, when targets and lures were equally common (PTP = 50%), MB was found to involve only a single criterion shift: The liberal male criterion was significant (*F*_1,39_ = 9.44, *p* = .004, *d*_*z*_ = 0.49), but the conservative female criterion was not (*F*_1,39_ = 3.90, *p* = .055, *d*_*z*_ = 0.31). Given an increased number of targets (PTP = 75%), the female target response criterion–but not the male criterion–reached the *adjusted* significance level (*c*, female targets: *F*_1,39_ = 9.15, *p* = .004, *d*_*z*_ = 0.48; *c*, male targets: *F*_1,39_ = 6.20, *p* = .017 > α, *d*_*z*_ = 0.39).

Bias statistics relating to *other-race* observers of 1000 ms stimuli are presented in Fig 5B. Observers were generally though marginally conservative when asked to target female hands. The group was slightly more inclined to report females as absent when targets and lures were equally prevalent (PTP = 50%: 0.10 ± 0.07), than when targets were rarer (PTP = 25%: 0.03 ± 0.07) or more common (PTP = 75%: 0.06 ± 0.09). When the task was to identify males, observers adopted more liberal response tendencies. The effect seemed to manifest particularly in the block containing 25% targets (-0.18 ± 0.09) relative to the ones containing 50% (-0.10 ± 0.06) or 75% targets (-0.09 ± 0.09).

Changes in *other-race* group bias across PTP conditions were found to be unsystematic. When observers searched for female hands, the average, conservative criterion changed neither as a linear (*F*_1,39_ = 0.12, *p* = .735, η^2^ < 1%) nor quadratic (*F*_1,39_ = 0.69, *p* = .411, η^2^ = 2%) function of PTP. When they searched for males, the group relaxed their criteria uniformly across PTP conditions (linear trend: *F*_1,39_ = 1.01, *p* = .322, η^2^ = 3%; quadratic: *F*_1,39_ = 0.48, *p* = .492, η^2^ = 1%). Lastly, although both female- and male-target criteria trended in the predicted, male-favouring direction, MB was found to be negligible whether PTP was 25% (*c*, female targets: *F*_1,39_ = 0.17, *p* = .682, *d*_*z*_ = 0.07; *c*, male targets: *F*_1,39_ = 3.80, *p* = .059, *d*_*z*_ = 0.31), 50% (*c*, female targets: *F*_1,39_ = 1.98, *p* = .167, *d*_*z*_ = 0.22; *c*, male targets: *F*_1,39_ = 2.64, *p* = .112, *d*_*z*_ = 0.26), or 75% (*c*, female targets: *F*_1,39_ = 0.41, *p* = .528, *d*_*z*_ = 0.10; *c*, male targets: *F*_1,39_ = 1.13, *p* = .294, *d*_*z*_ = 0.17).

#### Interim discussion

The data above support the prediction that observers–independent of their race or increased experience with own-race hands–are male-biased in their sex appraisals of those stimuli, but only when salient cues had been removed. Irrespective of long term experience (own-race vs. other-race observers), MB rates were uniform in spite of experiential changes in the short term (i.e. PTP). This finding fits the labile MB model presented in Fig 2A, and has some support from prior perceptual studies (e.g. [25, 39]).

Interestingly, asymmetries were found regarding the dual-mechanism explanation of MB [12]. Performance was weighted more strongly by the tendency to report males as present than to report females as absent, possibly because under uncertain conditions it might be costlier to miscategorise males than females [28, 29]. That said, the idea of risk minimisation under uncertainty does not explain why MB was found to be generally stronger for *more familiar* (own-race) stimuli, and particularly so in the case of Asian observers (Fig 4B: filled circles). This result is at odds with the negative link between expertise and MB documented in Wild et al.’s [26] face-based study. Moreover and more recently, Brielmann and Stolarova [36] reported that children have a proclivity to perceive amorphous drawings of adults as male; an effect that seems to weaken as ambiguity is reduced by contextual cues. In both cases, expertise was not defined in terms of race-based perceptual familiarity but rather cognitive development. That is, sex categorisation measures were either gathered exclusively from children [36], or compared between adult and child observers of adult and child faces [26]. Neither study was focussed on cross-race effects, and in the case of Wild et al. [26], only Caucasian stimuli were utilised in a deliberate move to avoid any own-race advantage (e.g. [47]). It is difficult then to speculate on whether such results should generalise to the present study of cross-race processing of adults, without at least replicating the latter (see Experiment 2, below).

### Experiment 2

As discussed, MB seems to arise in conditions in which sex cues are sufficiently difficult to discriminate. Limiting the time observers are allowed to process cues is one way to increase perceptual difficulty and thus amplify the effects of MB [12, 26]. Thus, a design identical to that of Experiment 1 is employed presently, but this time cues are limited to 125 ms presentations. A summary of the observer data collected in this experiment is tabulated in S3 Dataset.

#### Between-group analyses

**Fig 6:**
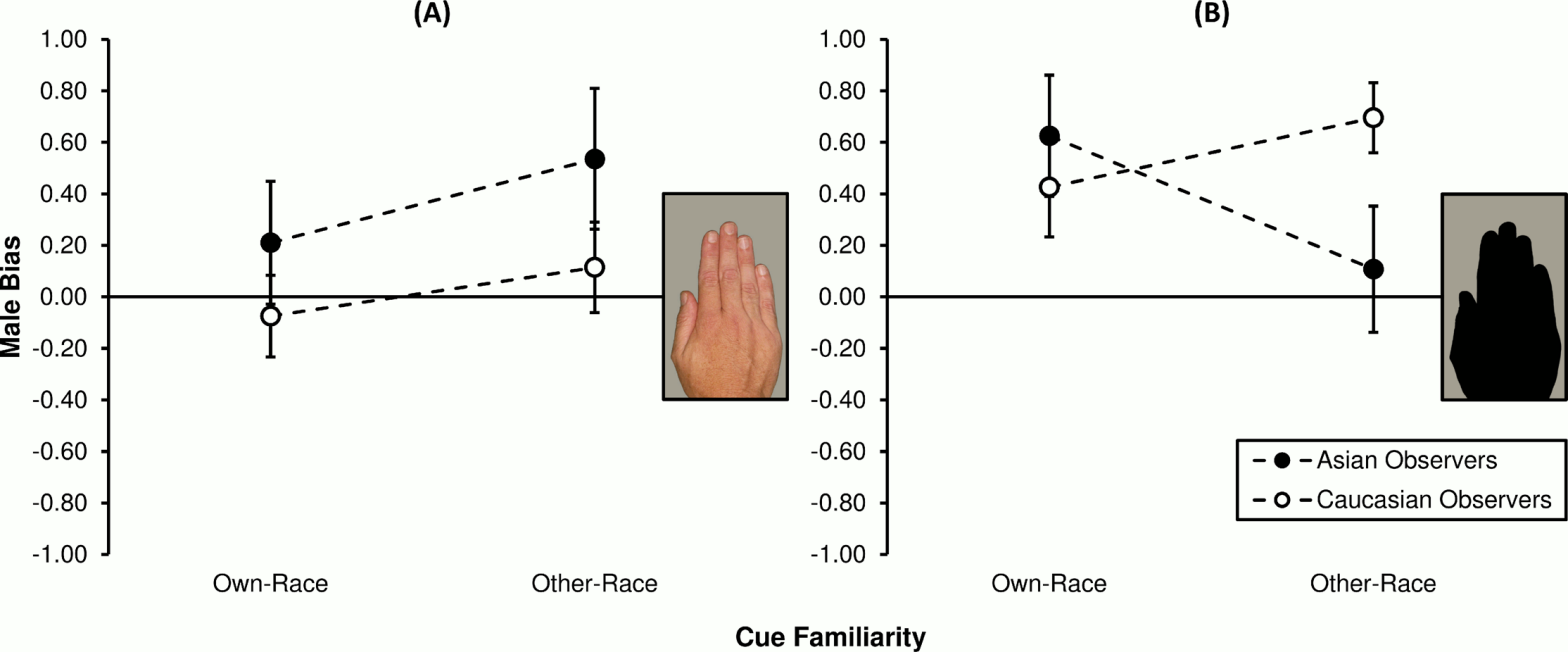
Sex classification bias for observers of hands each presented for 125 ms. Positive values represent, in standardised units, the mean tendency to report the presence of males *and/or* absence of females; negative values represent vice versa female bias (*c*_*A*_ = *c*_*female*_*—c*_*male*_). Performance is grouped by observer race (Asians or Caucasians) and cue familiarity (own-race or other-race hands), plotted separately for the (**A**) intact and (**B**) degraded visual cue condition. Vertical bars represent ±1 *SEM*.

Absolute MB (*c*_*A*_: *M* ± *SE*) for each group and hue/texture condition are plotted in Fig 6. When hue/texture cues were intact (panel A), observers of own-race hands (Asians: 0.21 ± 0.24; Caucasians: -0.07 ± 0.16) were less male-biased on average relative to other-race observers (Asians: 0.54 ± 0.27; Caucasians: 0.11 ± 0.18). Considering now the degraded cue condition (Fig 6B), this negative link between experience and bias was observed among the Caucasian group (own-race: 0.43 ± 0.19; other-race: 0.70 ± 0.14) though not the Asian group (own-race: 0.63 ± 0.24; other-race: 0.11 ± 0.24).

Planned orthogonal contrasts showed that when hand hue/texture was left intact, cue familiarity did not affect MB rates, irrespective of whether observers were Asian (*F*_1,152_ = 1.41, *p* = .238, η^2^ = 1%) or Caucasian (*F*_1,152_ = 0.47, *p* = .492, η^2^ < 1%). When hue/texture was absent from hand stimuli, familiarity was not found to mediate MB for either observer race group (Asian: *F*_1,152_ = 3.46, *p* = .065, η^2^ = 2%; Caucasian: *F*_1,152_ = 0.94, *p* = .334, η^2^ = 1%). Subsequent single-sample *F*-tests revealed that when colour and texture information was preserved, performance did not depart from the level expected were *H*_0_ true (own-race colour hands: *F*_1,39_ = 0.23, *p* = .637, *d*_*z*_ = 0.08; other-race colour hands: *F*_1,39_ = 3.94, *p* = .054, *d*_*z*_ = 0.31). By contrast, in the silhouette condition, both the 125 ms own-race (*F*_1,39_ = 12.11, *p* = .001, *d*_*z*_ = 0.55) and other-race (*F*_1,39_ = 7.57, *p* = .009, *d*_*z*_ = 0.44) marginal means were significantly male-biased.

#### Within-group analyses

**Fig 7:**
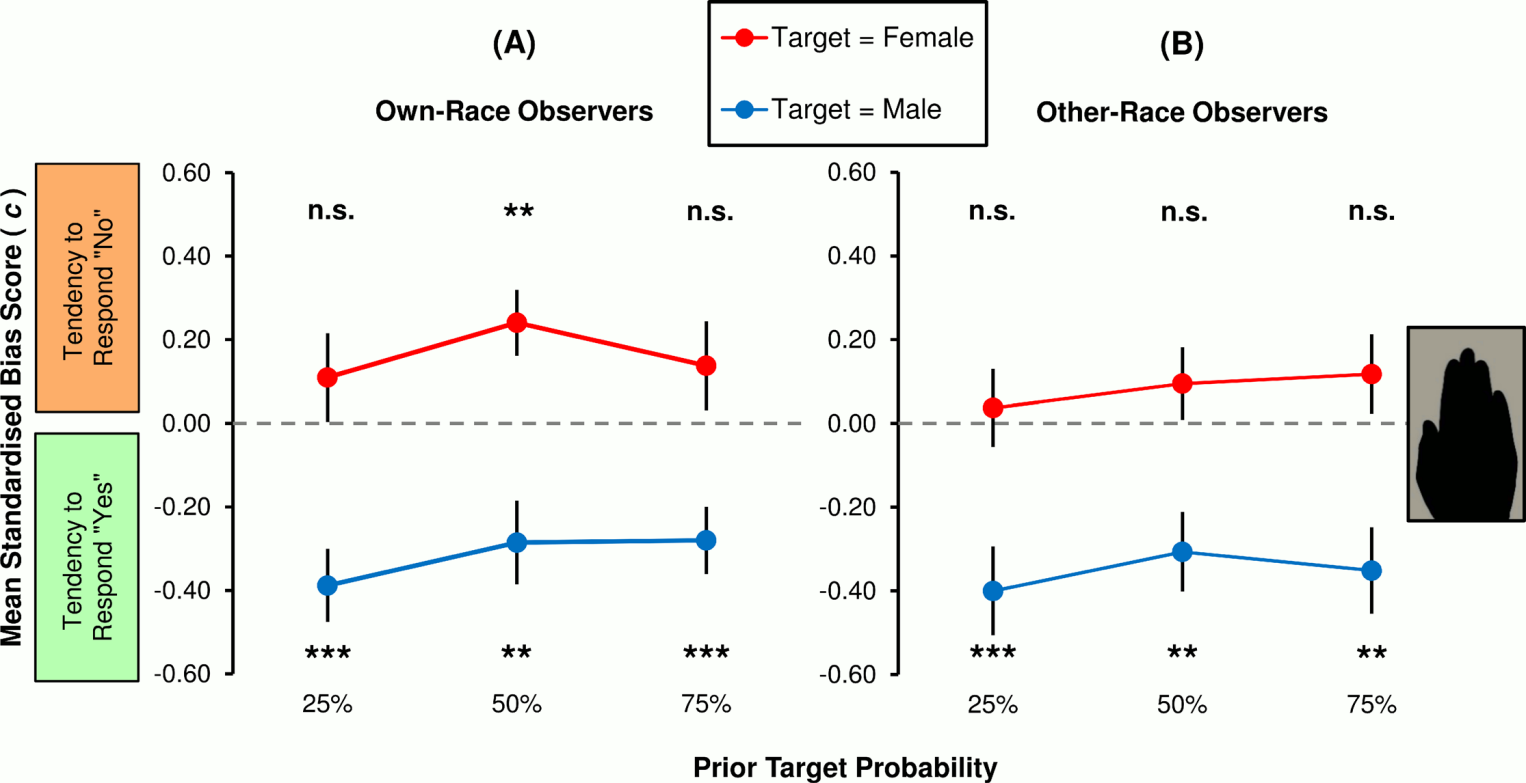
Group bias rates for brief (125 ms) presentations of silhouette hands. Performances in the ambiguous ‘silhouette’ condition are plotted separately for (**A**) own-race observers and (**B**) other-race observers. Means denoted *** and ** are significantly different from *c* = 0.00 at the .001 and .01 level, respectively. Means denoted * and ‘n.s.’ correspond to significance values .01 < *p* < .05 and *p* > .05, respectively, and therefore are not significant at the per-comparison criterion of α = .008. Vertical bars represent ±1 *SEM*.

Group bias scores for *own-race* hand observers are represented in Fig 7A. When observers’ task was to identify female hands, they showed a tendency to respond ‘no’–this varied in an inverted U-shape fashion relative to PTP (25%: 0.11 ± 0.11; 50%: 0.24 ± 0.08; 75%: 0.14 ± 0.11). In the male target task, the opposite tendency to make *positive* identifications diminished as PTP increased (25%: -0.39 ± 0.09; 50% -0.29 ± 0.10; 75%: -0.28 ± 0.08). *Other-race* observers’ performance (*c*: *M* ± *SE*) is summarised in Fig 7B. One can see that this group exhibited a slight tendency to respond ‘no’ when searching for female hands, which increased with the proportion of females (25%: 0.04 ± 0.09; 50% 0.09 ± 0.09; 75%:0.12 ± 0.09). The group also were inclined to respond ‘yes’ in search of males. Plotted against PTP, that tendency seemed to follow a quadratic trend: male affirmation bias was larger when there were less (25%: -0.40 ± 0.11) or more (75%: -0.35 ± 0.10) targets, relative to equal targets and lures (50%: -0.31 ± 0.10).

With respect to the *own-race* group (Fig 7A), planned contrasts showed that when target sex was female, bias did not differ linearly as a function of PTP (*F*_1,39_ = 0.10, *p* = .755, η^2^ < 1%). Moreover, despite the apparent curvilinear trend, analysis revealed that it was not significant (quadratic function: *F*_1,39_ = 3.60, *p* = .065, η^2^ = 8%). When males were defined as targets, mean bias was not subject to linear (*F*_1,39_ = 2.27, *p* = .140, η^2^ = 6%) nor quadratic (*F*_1,39_ = 0.61, *p* = .441, η^2^ = 2%) variation across PTP conditions. Single-sample *F*-tests showed that the dual-criterion MB is mediated by the relative frequency of targets to lures. In the 25% PTP condition, the male affirmation bias (*F*_1,39_ = 19.76, *p* < .001, *d*_*z*_ = 0.70) *but not* the female rejection bias (*F*_1,39_ = 1.06, *p* = .309, *d*_*z*_ = 0.16) was significantly different to optimality (i.e. *c* = 0.00). When PTP was 50%, the tendency to respond ‘male’ (*F*_1,39_ = 8.08, *p* = .007, *d*_*z*_ = 0.45) *and* ‘not female’ (*F*_1,39_ = 9.18, *p* = .004, *d*_*z*_ = 0.48) were also significant. Finally, with PTP set to 75%, the tendency to report presence of males (*F*_1,39_ = 12.08, *p* = .001, *d*_*z*_ = 0.55); but not the tendency to report females as absent (*F*_1,39_ = 1.65, *p* = .206, *d*_*z*_ = 0.20); was significant.

Attention is now turned to data sourced from *other-race* observers (Fig 7B). When target sex was female, group bias did not differ linearly across PTP conditions (*F*_1,39_ = 0.78, *p* = .383, η^2^ = 2%). Furthermore, bias was not found to track PTP via a quadratic path (*F*_1,39_ = 0.05, *p* = .824, η^2^ < 1%). Likewise when asked to target males, observers overall did not linearly (*F*_1,39_ = 0.51, *p* = .479, η^2^ = 1%) or quadratically (*F*_1,39_ = 0.80, *p* = .376, η^2^ = 2%) change their conservative criteria across blocks increasing in PTP. Further analyses showed that the pattern of MB in this group was characterised by a *single* criterion shift. That is, the male affirmation bias *but not* the female rejection bias was significant in all PTP conditions: 25% (*c*, male targets: *F*_1,39_ = 14.21, *p* = .001, *d*_*z*_ = 0.60; *c*, female targets: *F*_1,39_ = 0.16, *p* = .695, *d*_*z*_ = 0.06), 50% (*c*, male targets: *F*_1,39_ = 10.41, *p* = .003, *d*_*z*_ = 0.51; *c*, female targets: *F*_1,39_ = 1.19, *p* = .281, *d*_*z*_ = 0.17), and 75% (*c*, male targets: *F*_1,39_ = 11.50, *p* = .002, *d*_*z*_ = 0.54; *c*, female targets: *F*_1,39_ = 1.53, *p* = .224, *d*_*z*_ = 0.20).

#### Interim discussion

Outcomes from Experiment 2 showed that MB was more pronounced as a result of the limited exposure time (125 ms) relative to Experiment 1 (1000 ms). Again, own- but not other-race observers were (at least under certain PTP conditions) liberal identifiers of males and also conservative identifiers of females, though the male ‘yes’ bias was stronger than female ‘no’ bias. In contrast, other-race observers were only significantly biased toward identifying males and not against identifying females. Furthermore, the labile MB hypothesis (see Fig 2A) has been supported; both own- and other-race observers update their MB criteria relative to each PTP condition, suggesting that MB is updated on the basis of live perception. In other words, simply providing more female cues does not inflate MB, nor does it deflate MB.

### Convergent evidence

The bias outcomes documented in Gaetano et al.’s study [12] and above in the present study were all invoked via hand stimuli developed especially by the first author. Follow-up experiments were designed to probe whether comparable effects would arise in relation to a different stimulus set, as developed by other researchers. Specifically, Gaetano et al.’s [12] within-group design was replicated to further test the idea that sex perception employs common behavioural responses regardless of the dimorphism used or instructions provided to observers. Here then, it was predicted that the dual-criterion MB [12] would also arise when ambiguous *facial representations* are utilised in place of hands. It was also predicted that observers would perceive female face profiles as androgynous more often than male exemplars.

#### Method

First year SCU psychology students aged 18 or over voluntarily participated in either of two pilot experiments, hereby called *demonstrations*. Twenty-five individuals (19 female) were recruited to participate in Demonstration 1; a separate cohort of 44 (33 female) participated in Demonstration 2. Stimuli were 30 adult (15 female) face profiles with neutral expressions, derived from the Stirling face set (*Psychological Image Collection at Stirling*: http://pics.stir.ac.uk) and manipulated such that each resembled a black silhouette shape on uniform grey background. The set was copied to produce identical leftward and rightward facing versions. Observers were provided with a URL to access the experiment, which they could complete on-campus in a computer laboratory or via undefined apparatus (e.g. home laptop). Observers could choose between two input methods: computer keyboard or onscreen buttons.

The sequence of each trial was almost identical to that described in the main experiments. The one exception was the response screen, which in these demonstrations was presented indefinitely until a response was made. In *Demonstration 1*, the 60 profile images were randomly sequenced within each of four blocks: two presentation durations (125 ms, 1000 ms) and two target sexes (female, male). The observer’s task was to indicate whether each silhouette face profile portrayed a (female or male) ‘target’ or ‘not’. In *Demonstration 2*, the same stimuli were shown to a separate group, in random order, once per presentation duration block (ms: 125, 250, 500, 1000). This time, observers were asked to indicate whether each image was ‘androgynous’ or ‘not’. Block order was counterbalanced across observers, as was the key/button alternatives associated with each response. Performance was measured as a function of presentation duration, and target or stimulus sex. Data obtained from both demonstrations are tabulated in S4 Dataset.

#### Demonstration 1

**Fig 8:**
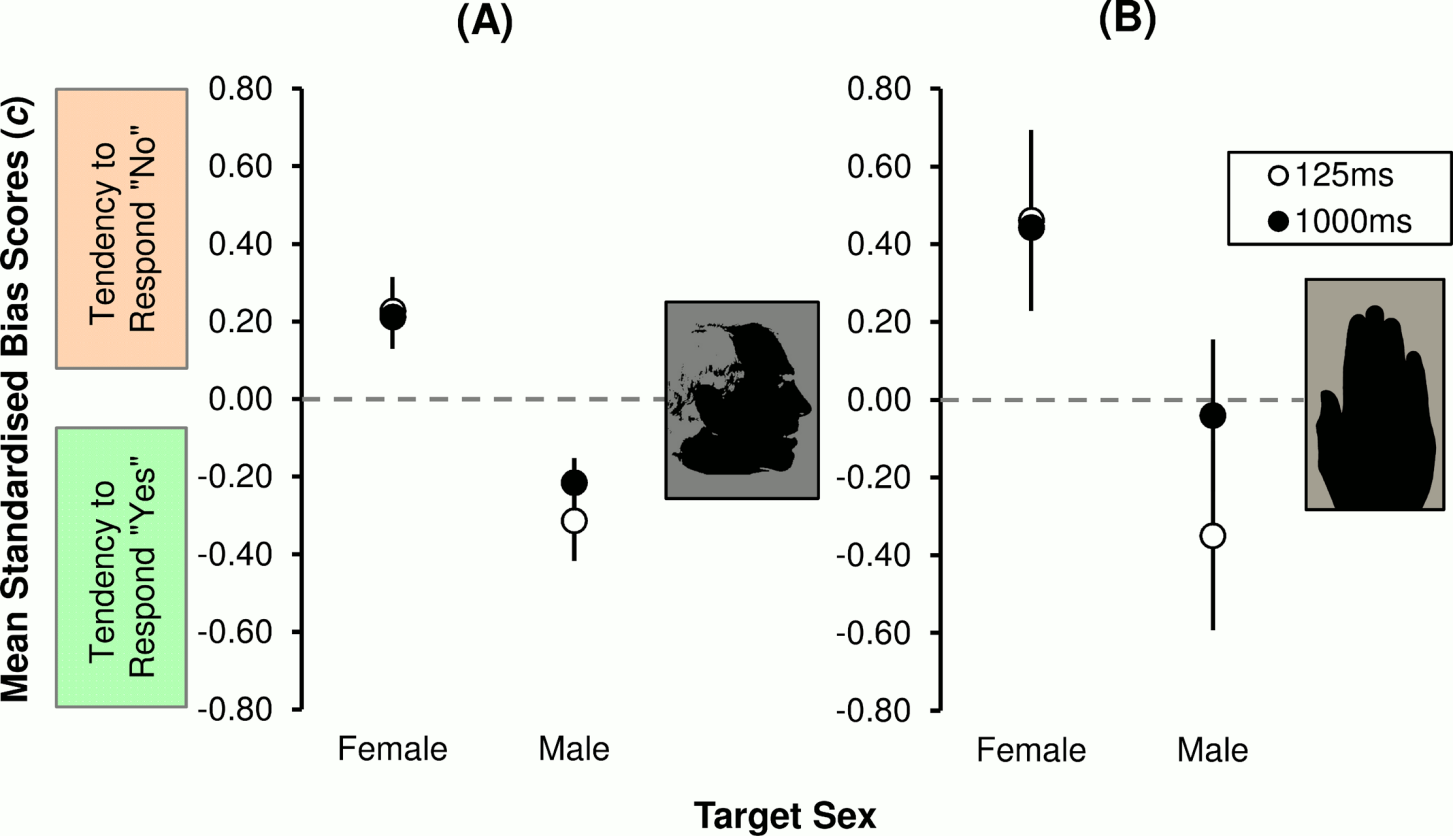
Caucasian observer sex discrimination bias for own-race hand images. Standardised bias (*c*) scores corresponding to two Caucasian, own-race observer groups: (**A**) silhouette face observers from the current study, and (**B**) silhouette hand observers from Gaetano et al. [12]. Performance is plotted as a function of target sex (female, male) and presentation duration (125 ms, 1000 ms); in each condition, there was an equal prior target probability, and hands were presented without hue or texture information. Vertical bars represent ±1 *SEM*.

The core prediction examined here was that the dual-criterion MB [12] would manifest when ambiguous facial representations are utilised in place of ambiguous hands. Observer bias statistics (*M* ± *SE*) are described in text and also summarised in Fig 8A. For comparison and later discussion, bias data from the Gaetano and colleagues’ previous hand-based study [12] are replicated in Fig 8B. When observers were targeting female *face profiles*, bias was mostly static across presentation duration blocks (125 ms: 0.23 ± 0.09; 1000 ms: 0.21 ± 0.08). When observers were searching for male targets, presentation duration seemed to have a larger effect on bias (125 ms: -0.31 ± 0.10; 1000 ms: -0.22 ± 0.06). Together, observers were both conservative when distinguishing females from non-females, and liberal when distinguishing males from non-males.

Subsequent contrasts revealed that observers were more affirmative when targeting males relative to females (*F*_1,24_ = 11.17, *p* = .003, η^2^ = 32%). Nonetheless, response bias did not change overall as a function of presentation duration (*F*_1,24_ = 0.86, *p* = .362, η^2^ = 3%) nor was an interaction detected between target sex and presentation duration (*F*_1,24_ = 1.03, *p* = .321, η^2^ = 4%). Thus, presentation duration was collapsed, and the marginal means of target sex were compared against the null criteria value. As a result, a dual-criterion MB was exposed; observers had tighter criteria for female targets (*F*_1,24_ = 7.87, *p* = .010) *and* looser criteria for male targets (*F*_1,24_ = 11.82, *p* = .002), with the latter effect being the larger of the two (*d*_*z*_, females = 0.56; *d*_*z*_, males = 0.69).

#### Demonstration 2

The relationship between degree of stimulus fe/maleness and subsequent ratings of stimulus sex demonstrably follows a cumulative Gaussian curve that is shifted, such that the point of subjective androgyny is slightly female [25, 48]. A resulting prediction is that when sex cues are difficult to perceive, stimuli will overall appear more male. Because of the shift in the psychometric function, female stimuli should appear more androgynous (less female) and male stimuli should appear less androgynous (more male). It is hereby predicted that more female relative to male images will be reported as androgynous, at least at the briefest (most difficult) presentation duration.

**Fig 9:**
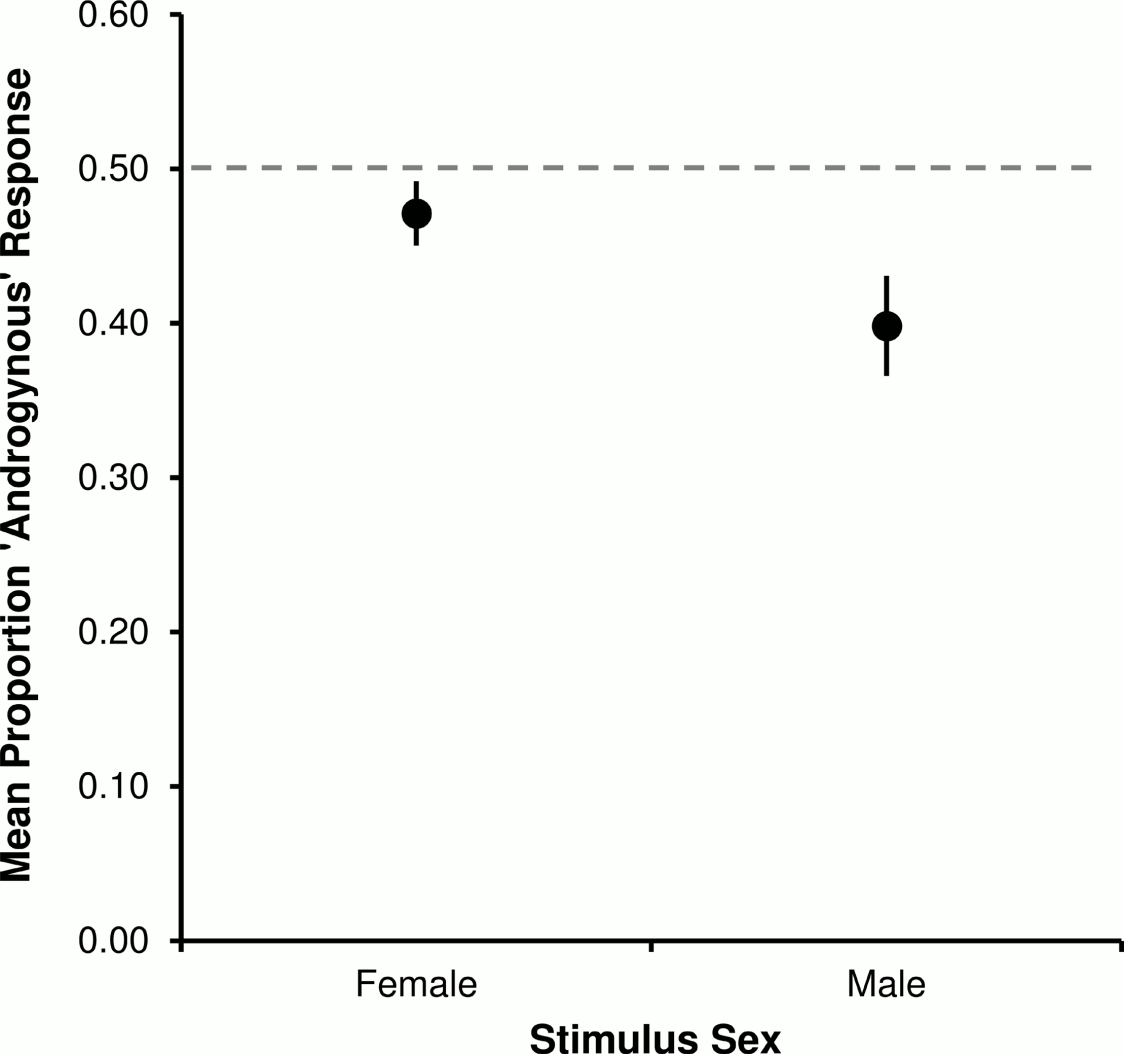
Average rate of reporting silhouette face profiles as androgynous. Data are plotted as a function of stimulus sex (female and male), and were averaged across four stimulus exposure times (125 ms, 250ms, 500ms, and 1000 ms). The horizontal dashed line represents the rate expected by random responding; vertical lines represent ±1 *SEM*.

Overall, observers were more likely to label female than male silhouette face profiles as androgynous. In terms of group proportion of ‘androgynous’ responses, that trend was apparent at each presentation duration: 125 ms (female: 0.47 ± 0.03; male: 0.41 ± 0.03), 250ms (female: 0.49 ± 0.02; male: 0.40 ± 0.03), 500ms (female: 0.46 ± 0.03; male: 0.41 ± 0.04), and 1000 ms (female: 0.47 ± 0.02; male: 0.38 ± 0.03). Planned contrasts inferred no linear relationship between presentation duration and the rates at which face profiles were categorised as androgynous (female profiles: *F*_1,43_ = 0.23, *p* = .638, η^2^ < 1%; male profiles: *F*_1,43_ = 1.49, *p* = .230, η^2^ = 3%). As predicted though, there was an overall effect of stimulus sex (*F*_1,43_ = 8.03, *p* = .007, η^2^ = 16%); female face profiles were categorised to be androgynous relatively more often than were male face profiles. The marginal means of stimulus sex (females: 0.47 ± 0.02; males: 0.40 ± 0.03) are depicted in Fig 9.

#### Interim discussion

Taken together, the preliminary results here provide additional support for the notion that MB is indeed a ubiquitous perceptual phenomenon. By way of comparison to Gaetano et al.’s [12] initial study, observers are both conservative when identifying females, and liberal when identifying males. Similar trends can be seen across experiments; whether silhouette hands or faces are shown, observers tend to be more male-biased when viewing time is limited (125 ms), especially if they are searching for *male* targets. In absolute terms, criteria for hands measure larger than criteria for faces, with one surprising exception: given a longer viewing time (1000 ms), liberal male criteria for hands on average are much smaller than those for faces. This is probably not a true effect, but an artefact of the smaller sample size in the previous hand-based study (*n* = 11 [12]). In fact, under similar conditions (i.e. silhouette hands, PTP = 50%) in the larger, main study described above, the group male criterion was *significantly* liberal. Another point is that there is more variance around the means calculated in the smaller hand-based study, relative to those derived from the current face-based demonstration. In essence, outcomes converge to suggest the existence of a dual-criterion MB that is stimulus invariant, which arises when the prior probability of females or males does not deviate from the norm.

The other major finding to arise from these preliminary data is that MB is better explained by perception than by task demands. Not only can it arise under different sex categorisation tasks (e.g. [12, 24, 48, 49]), present data show it can even arise when observers are asked to categorise something other than sex. Consistent with the notion that cues within the ambiguous range of the female-male spectrum are perceptually male-shifted [25, 48], observers were more likely to categorise ambiguous *female* profiles as androgynous.

In sum, the preliminary findings here converge on the theory that under conditions of uncertainty, sex perception is characterised by male-biased behaviour that can manifest independent of stimulus or task. Of course, to become more confident of that assertion the data presented here require replication under a wider range of conditions. Nevertheless, the preliminary findings open new lines of enquiry for future research. Most notably, the role of perceptual expertise- and race-invariance could be explored using silhouette face profiles as per the main Methods. Finally, if increased ‘androgynous’ categorisations of females is truly a product of ambiguity, then replicating Demonstration 2 with clearly dimorphic (i.e. colour) stimuli should negate the effect.

## General Discussion

The general objective of this work was to test the robustness of MB under a range of different conditions, with a specific eye to testing its invariance relative to long- and short-term experience. It was specifically predicted that given ambiguous viewing conditions, observers would: (i) categorise sex from hands in a male-biased manner, irrespective of race-based expertise; (ii) adopt both a strict criterion when assigning a target as female, and a loose criterion when assigning a target as male, and; (iii) fix those criteria independent of the weight of target relative to lure cues in the short term. Results showed, as expected, that Caucasian and Asian observers’ sex categorisations were overall male-biased, and were so regardless of the race of hands viewed. Further, MB arose predominately as a tendency to respond ‘male’ rather than a tendency to respond ‘not female’, although the dual-criterion account was partially supported on inspection of own-race bias measures. Finally, bias measures did not change linearly by PTP, suggesting that MB is not determined simply by an increased incidence of males over time.

Collectively, these findings could have applications in not just the sex perception literature but also more diverse fields of enquiry. For instance, they contribute new knowledge regarding the evolved functions of human social vision. This study is the first to show that MB is pan-cultural and characterises the sex judgements of many individuals–the frequency of which outweighs the number of female- (or other-) biased cases (see Fig 3). Indeed, such an ubiquitous tendency to ‘err on the side of male’ if unsure is surprising given that the human species, unlike other primate species (e.g. baboons [50]), have near-equal and stable proportions of females and males. MB presumably, therefore, serves a purpose unrelated to sexual accessibility at the population-level ([27]; cf. [51]), and the one discussed further below is *risk detection*.

The other potentially important application of this research is in the area of forensic psychology. Numerous studies (e.g. those meta-analysed in [52]) have shown how witnesses are liable to make potentially grave mistakes in the identification of perpetrators who have a different ethnic background. Forensic identifications are often made on the basis of incomplete or low fidelity evidence, such as low frame rate security footage. The present research may add clarity to otherwise fuzzy identifications; it implies that the worth of witness statements about the sex or race of perpetrators should be offset by a best estimate of bias, depending on the quality of both initial witness conditions and subsequent evidence (e.g. handprints: [53]). Indeed, these factors could inform a quantitative model that would complement the process of determining a suspect’s characteristics in the absence of certainty. For instance, the probability of ‘male suspect present’ witness reports is presumably high given the lay assumption that males are trialled for crimes more commonly than are females: *p*(report: male = guilty | prediction: male = guilty). By taking account of ambiguity in crime scene environments (and thus, by estimating the likelihood of MB in each case), investigators could determine the inverse probability: that a male is guilty of a crime, given the report of such: *p*(prediction: male = guilty | report: male = guilty). Therefore, it is posited that law enforcement agents can reduce their rate of false leads by developing an understanding of MB and other-race effects in particular, and social categorisation processes under uncertainty in general.

### The role of perceptual ambiguity and observer familiarity

The ambiguity dependence of MB documented elsewhere (e.g. [11]) has been consistently replicated. Assisted by colour and shading cues, observers are generally no more prone to perceive a male hand as female than they are to make the opposite mistake. However, eliminating those cues and/or limiting the signal processing time not only reduces sex categorisation sensitivity, but also results in an absolute criteria shift which favours the identification of male targets or the omission of female targets.

Taking a wider look at the literature, it becomes apparent that all extant accounts of MB have been qualified by perceptual ambiguity. What all of those studies (including [12]) have in common is that race was never included as a variable of interest; observers were either described as Caucasian, or were recruited as students at a predominately Caucasian university. By contrast, observer race differed across groups in the present study, and was found to have no impact on observers’ MB rates on the whole. That MB is not Western-specific suggests that it is not purely the result of enculturation but rather a perceptual process shared by members across cultures.

Bias did not diverge overall by observer race, but partial divergence was exhibited as a function of observer *familiarity*. Caucasian and Asian observers who were afforded 125 ms to view own-race (more familiar) or other-race (less familiar) hands were overall significantly male-biased. At the longer viewing time though (1000 ms), only observers of *more familiar* hands were significantly male-biased. This is unexpected in light of studies exemplifying a perceptual advantage for own-race stimuli (as reviewed by [52]; cf. [54]), but might be explained by a general inability to encode other-race stimuli presented at longer durations. A large-scale appraisal of the cross-race face recognition literature has revealed that memory is not merely poorer for other-race faces, but also there is a higher tendency to judge such as ‘seen before’ [52]. Within that literature, studies tend to involve stimulus presentations lasting in excess of 1000 ms (e.g. [55, 56]), presumably to allow enough time for exemplars to be memorised. Thus, it is possible that the general memory disadvantage for other-race stimuli may explain the lack of sex categorisation bias seen here, at least given sufficient presentation time. On the contrary, at 125 ms per stimulus, memory is less likely to be a factor in the observer’s criteria for other-race females and males.

### Qualifiers and constants of the dual-criterion male bias

Breaking MB down into target sex-specific components, current data shows a proclivity for male identifications which is accompanied by *and yet stronger than* a disinclination for female identifications. This seems to agree with a general hypothesis put forth in the Introduction, that there is an evolutionary incentive to, when uncertain, miss detecting as few males as possible. A general caveat that challenges the application of current data to the risk minimisation hypothesis is that the latter is informed mostly by the face and body perception literature. The hand perception literature is, by comparison, small and new. Although the observed hand-based MB is argued to support the risk minimisation hypothesis, further tests are required to show that hands are relevant stimuli in the way that faces and bodies are. One test would be to prime observers with images of violent scenes, to elicit a MB from hands versus other stimuli. Another possible test is currently underway, which involves presenting observers with pairs of female and male hands, and recording eye movements. The latter test should reveal whether there is an attentional component to MB, independent of task demands associated with asking observers to target females or males.

#### Own-race observations

As was the case for the Caucasian observers of Caucasian hands in a previous study [12], current *own-race* observers shifted both their male and female target criteria in the predicted directions. That is, the own-race group (of which half consisted Asian observers) were both more liberal when searching for male hands, and more conservative when looking for females. Under all PTP conditions and irrespective of presentation duration, the liberal male criterion was significant. With regard to the conservative female criterion however, significance was reached under select conditions only. If own-race hands were presented for 125 ms, both criteria shifts were significant when PTP was *equal*; if instead hands were shown each for 1000 ms, the dual shift reached significance when PTP was *unequal*.

A follow-up experiment using own-race silhouette face profiles as stimuli revealed the same significant pattern, at both presentation durations, and with *equal* PTP (see Demonstration 1). In conjunction with the main study outcomes, own-race observers of ambiguous, diverse representations (i.e. hands, face profiles) exercise hesitance when reporting the presence of females, and also a willingness to report the presence of males, yet the symmetry of this effect is broken by deviating frequencies of female or male targets across blocks. That is, the male *but not female* target criterion shift appears robust despite such PTP deviation. Finally, a second pilot experiment using silhouette face profiles demonstrated that own-race observers can exhibit MB in lieu of explicit instruction to categorise sex (Demonstration 2). This is concordant with the working prediction that MB mechanisms are generally perceptual: sufficiently noisy female cues will not meet the observer’s inclusion criteria, and hence will tend to be regarded as ‘male’, ‘not female’, ‘androgynous’, etc.

Other-race observations. The dual-criterion MB trend also emerged among other-race observers, but the criteria shifts did not always depart from zero. If hands were shown for 125 ms, only the liberal male criterion was significant, and was so across all PTP conditions. If hands were shown for 1000 ms, both criteria shifts failed to reach significance, irrespective of whether targets were equal in proportion to, or rarer or more common than lures. Taking a wider view, MB in relation to the less familiar other-race hands is dominated by a liberal male criterion, and only if exposure times are brief. Even though observers were presumably naïve regarding the race of hands presented to them, it is possible that the less familiar other-race hands were implicitly regarded as members of an out-group.

Removed from the familiar context of an in-group, an individual may be justified in prioritising the search for sources of physical threat. Indeed, other-race faces not only involve distinct attentional strategies [17, 20, 57]; they also can evoke enduring and physiological correlates of fear from the observer [3, 32–34]. Further, the tendency to use force [4] or act defensively [3] with regard to other-race agents is apparently qualified by perceptions of sex–that is, males more than females tend to attract such primitive, fight/flight responses. This type of interaction captures neatly the asymmetric bias documented in the present study; observers showed a willingness to accept other-race hand shapes as male and yet showed no resistance to accepting them as female. The same type of interaction may account for observers who exhibit a tendency to judge *male* point-light walkers as *advancing* (and female figures as retreating) when there are no cues to indicate either course ([30, 58, 59]; cf. [60]). In an unfamiliar environment, the ‘male advancing bias’ would represent a sensible risk avoidance tactic, yet to date this has not been explored using own- and other-race point-light figures. Nonetheless in the present study, ambiguous presentations of other-race hands resulted in a curious pattern of MB: as if missing male targets was an adverse outcome, yet mistaking females for males was of no consequence.

## Conclusion

In conclusion, MB is an ambiguity-dependent perceptual effect which arises when an observer must discriminate sex from degraded cues or under increased duress. This study extends on what was previously known about the parameters and function of this effect. First of all, MB per se is not specific to Western observers, nor is it dependent absolutely on the observer’s familiarity with certain race cues. Dividing this general pattern into target-specific criteria, it seems a dual-criterion MB arises under certain conditions for own-race observers, but not for other-race observers. In relation to own-race observers, the predicted dual shift in criteria is, to some degree, stimulus- and task-invariant–observers of hand *and* face silhouettes both tend to tighten their criteria for females and loosen their criteria for males, independent of the task assigned to them. Furthermore, MB is apparently not just a rote response strategy, but may be adaptively updated in accordance with varying target-to-lure ratios.

Overall, and in keeping with the theory that sex perception is a dynamic and interactive component of processing whole individuals [61], the apparently pan-stimulus nature of MB implies that its representation in cortical space is not specific to just face-selective areas–it is interesting then to speculate on a population of stimulus invariant, sex-tuned neurons that are mediated by expertise, which distribute information via different pathways depending on whether male or female cues surpass certain thresholds. At the least, this study proposes that MB exists across cultures and has value as a risk-avoidance heuristic; consequent investigations will benefit more applied areas such as forensics and law.

## Supporting Information

S1 Dataset Preliminary analyses of observer age covariance. See Methods in main text.
(XLSX)

S2 Dataset Data and analyses pertaining to Experiment 1. Between- and within-group analyses are shown on separate worksheets.
(XLSX)

S3 Dataset Data and analyses pertaining to Experiment 2. Between- and within-group analyses are shown on separate worksheets.
(XLSX)

S4 Dataset Additional measures of male bias. See Convergent Evidence in main text.
(XLSX)
